# Panaxydol attenuates ferroptosis against LPS-induced acute lung injury in mice by Keap1-Nrf2/HO-1 pathway

**DOI:** 10.1186/s12967-021-02745-1

**Published:** 2021-03-02

**Authors:** Jiucui Li, Kongmiao Lu, Fenglan Sun, Shanjuan Tan, Xiao Zhang, Wei Sheng, Wanming Hao, Min Liu, Weihong Lv, Wei Han

**Affiliations:** Qingdao Municipal Hospital, School of Medicine, Qingdao University, No. 1, Jiaozhou Road, Qingdao, 266011 Shandong China

**Keywords:** Panaxydol, Ferroptosis, Acute lung injury, LPS, Inflammation

## Abstract

**Background:**

Acute lung injury (ALI)/acute respiratory distress syndrome (ARDS) induces uncontrolled and self-amplified pulmonary inflammation, and has high morbidity and mortality rates in critically ill patients. In recent years, many bioactive ingredients extracted from herbs have been reported to effectively ameliorate ALI/ARDS via different mechanisms. Ferroptosis, categorized as regulated necrosis, is more immunogenic than apoptosis and contributes to the progression of ALI. In this study, we examined the impact of panaxydol (PX), isolated from the roots of Panax ginseng, on lipopolysaccharide (LPS)-induced ALI in mice.

**Methods:**

In vivo, the role of PX on LPS-induced ALI in mice was tested by determination of LPS-induced pulmonary inflammation, pulmonary edema and ferroptosis. In vitro, BEAS-2B cells were used to investigate the molecular mechanisms by which PX functions via determination of inflammation, ferroptosis and their relationship.

**Results:**

Administration of PX protected mice against LPS-induced ALI, including significantly ameliorated lung pathological changes, and decreased the extent of lung edema, inflammation, and ferroptosis. In vitro, PX inhibited LPS-induced ferroptosis and inflammation in bronchial epithelial cell line BEAS-2B cells. The relationship between ferroptosis and inflammation was investigated. The results showed that ferroptosis mediated inflammation in LPS-treated BEAS-2B cells, and PX might ameliorate LPS-induced inflammation via inhibiting ferroptosis. Meanwhile, PX could upregulate Keap1-Nrf2/HO-1 pathway, and selective inhibition of Keap1-Nrf2/HO-1 pathway significantly abolished the anti-ferroptotic and anti-inflammatory functions of PX in LPS-treated cells.

**Conclusion:**

PX attenuates ferroptosis against LPS-induced ALI via Keap1-Nrf2/HO-1 pathway, and is a promising novel therapeutic candidate for ALI.

## Background

Acute respiratory distress syndrome (ARDS) is a major cause of respiratory failure and one of the most challenging clinical conditions. It is characterized by the rapid and intense inflammatory response due to the activation of pro-inflammatory and oxidant pathways, resulting in the accumulation of neutrophils, interstitial edema, and the injury of the alveolar epithelium in the lung tissues, followed by pulmonary extracellular matrix (ECM) remodeling [1]. It has high morbidity and mortality rates with more than 3 million ARDS cases and 75,000 deaths annually worldwide [2]. For the past few years, numerous studies have attempted to find a specific drug to reduce the high mortality rate of ARDS patients, but there is still a mortality rate of ~ 40% in intensive care units [3, 4]. Acute lung injury (ALI), a less severe form of ARDS, is also a disorder of acute inflammation. There is a great deal of research indicating that gram-negative bacterial infection is one of the most important causes of ALI, and lipopolysaccharide (LPS), the major component of outer membranes of gram-negative bacteria, can cause the lung injury and inflammatory response [5, 6]. In fact, LPS-induced ALI in mice has been a well-accepted model for investigating ARDS because it mimics pathological events such as the inflammatory and histological changes observed in this disease [7, 8].

Ferroptosis, a distinct form of regulated necrosis, is distinct from apoptosis, autophagy, and other forms of cell death, and is an iron- and lipid hydroperoxide-dependent cell death [9]. It is involved in all kinds of human diseases, and its inhibition could diminish the clinical symptoms in experimental models of neurodegeneration, liver injury, renal failure and heart injury [10–13]. Recently, increasing studies have shown that ferroptosis occurs in ALI, and its inhibition is effective in alleviating the disease. For example, Li et al. [14] reported that ferroptosis contributes to intestinal ischemia/reperfusion (I/R)-induced ALI, and iASPP treatment inhibits ferroptosis and alleviates intestinal I/R-induced ALI partly via Nrf2; Dong et al. [15] found that Nrf2 inhibits ferroptosis and protects against I/R-induced ALI via regulating SLC7A11 and HO-1; Qiu et al. [16] demonstrated that Nrf2 protects against seawater drowning-induced ALI via inhibiting ferroptosis; and Liu et al. [17] reported that ferrostatin-1 (Fer-1, ferroptosis inhibitor) alleviates LPS-induced ALI via inhibiting ferroptosis. It is clear from these studies that Nrf2 is an important negative regulator of ferroptosis in ALI; ferroptosis contributes to the progression of ALI; and ferroptosis inhibition by activation of Nrf2 provides a novel therapeutic target for ALI, which tempt us to identify some drugs that activate Nrf2 and inhibit ferroptosis against ALI.

Panax ginseng, a well-known medicinal plant, has long been used in the traditional medicine in far eastern countries such as China, Korea and Japan for detoxification, controlment of blood glucose levels, prevention of arteriosclerosis, and anti-aging [18–20]. Polyacetylene compounds are the main components of pharmacological action of Panax ginseng. Panaxydol (PX), as one of the most widely studied polyacetylene compounds, possesses various biological activities, such as antifatigue activity, antitumor activity, and neurodegenerative protection activity [21–23]. In addition, recently, Guo et al. [24] reported that PX could suppress aristolochic acid-induced renal failure by suppressing oxidative stress through activation of Keap1-Nrf2 signaling pathway. But whether and how PX plays the pharmacological role in the treatment of ALI has not been reported. Thus, the present study aimed to explore whether PX inhibits ferroptosis and protects against LPS-induced ALI in vivo and in vitro via  activation of Keap1-Nrf2 pathway.

## Materials and methods

### Animals

Specific pathogen-free (SPF) male C57BL/6 mice (6–8 weeks old, 20–24 g body weight) were purchased from the Experimental Animal Center, Anhui Medical University (Hefei, China), housed in cages, given free access to food and water, and used after 2 weeks of quarantine and acclimatization. All procedures involving animals were approved by the Animal Ethics Community of Qingdao Municipal Hospital. All surgeries were performed under sodium pentobarbital anesthesia, and all efforts were made to minimize suffering.

### Murine model of LPS-induced ALI

All mice were randomly divided into five groups (8 mice every group): control group, LPS group, PX+LPS group (administered 20 mg/kg PX), Fe+LPS group (administered 15 mg/kg Fe-citrate (III)), and PX+Fe+LPS group (administered 20 mg/kg PX and 15 mg/kg Fe-citrate (III)). Fe-citrate (III) (Fe; Sigma-Aldrich, St. Louis, MO, USA) was dissolved in stroke-physiological saline solution (SPSS). PX (ChemScene LLC, South Brunswick Township, NJ, USA) was dissolved in dimethyl sulfoxide (DMSO; Sigma-Aldrich), and further diluted in SPSS. Intravenous injection of Fe or/and intraperitoneal injection of PX were performed from day 0 to day 2. At 1 h after the final Fe and PX treatment, the mice were anesthetized with 30 mg/kg of pentobarbital sodium (Beijing Chemical Co., China) and then LPS (10 µg/mouse; InvivoGen, San Diego, CA, USA) or SPSS was injected into the trachea. After administration, the mice were placed in a vertical position and slowly shaken for 1 min to ensure LPS or SPSS distributed evenly between the left and right lungs. Mice were sacrificed at 24 h post LPS stimulation.

### Collection of bronchoalveolar lavage fluid (BALF) and inflammatory cell counting

According to our previous report [25], the thoracic cavity was opened, and the right lung at the mainstem bronchus was tied off. BALF was collected by cannulating and lavaging left lung three times with 1.0 ml of PBS. Then BALF was centrifuged for 10 min at 300×*g*, and the cell pellet and supernatant of BALF were collected separately. Then the cell pellet was resuspended in PBS, and total white blood cells (WBCs) were counted by using a hemocytometer (Hausser Scientific Co, Horsham, PA, USA). The neutrophils in BALF were stained with Wright (Sigma-Aldrich), and counted by 200 cells/slide.

### Measurement of pro-inflammatory cytokines in BALF or cell culture supernatants

The BALF supernatant was collected and stored at − 80 °C. Cells were cultured under the indicated conditions, and culture supernatants were collected. Then the pro-inflammatory cytokines TNF-α, IL-1β, and IL-6 level were determined in BALF and cell culture supernatants via ELISA kits (Bender Medsystems, Burlingame, CA, USA).

### Measurement of BALF protein concentration

The concentration of BALF protein was detected by using the bicinchoninic acid (BCA) protein assay kit (Beyotime, Shanghai, China) according to the manufacturer’s instructions.

### Determination of myeloperoxidase (MPO) activity

To quantify neutrophil infiltration, MPO activity in the homogenized lung tissues was evaluated by using a MPO Detection Kit (Nanjing Jiancheng Bioengineering Institute, Nanjing, China). Briefly, lung samples were homogenized and then centrifuged at 12,000*g* at 4 °C for 20 min. Subsequently, the supernatants were collected. MPO activity in supernatants was determined at 460 nm using a 96-well plate reader, and presented as units per gram of total protein (U/g). Total protein concentrations were calculated via the BCA protein assay kit.

### Lung wet/dry (W/D) weight measurement

The severity of pulmonary edema was assessed by the W/D ratio. Briefly, after mice sacrifice, lung tissues were removed and immediately weighted for wet weight (W). Then the wet lung tissues were placed in an oven at 60 °C for 48 h, and weighted again to obtain the dry weight (D). Next, the W/D ratio was calculated.

### Haematoxylin–eosin (HE) staining

The right lower lung of each mouse was fixed in 10% neutral buffered formalin for 24 h. Then lung tissues were dehydrated, embedded in paraffin, sectioned at 3-μm thickness on a rotary microtome, and stained with hematoxylin and eosin (H&E) to analyze the pathological alterations of the lung tissues. Lung injury was assessed from four categories: interstitial inflammation, neutrophil infiltration, congestion and edema. A score of 0 to 4 was used to describe the severity of every category, with 0 representing minimal damage; 1 mild damage; 2 moderate damage; 3 severe damage; and 4 very severe damage. Lung injury score was the sum of the individual scores for every category.

### Measurement of the biomarkers of ferroptosis

The biomarkers of ferroptosis Fe^2+^, malondialdehyde (MDA) and glutathione (GSH) level, and Glutathione Peroxidase 4 (GPX4) activity in lung tissues and cells were assessed using the Iron Assay Kit (Sigma-Aldrich), MDA Assay Kit (Sigma-Aldrich), GSH assay kit (Sigma-Aldrich), and GPX4 ELISA kit (Wuhan USCN Business CO, Ltd. China), respectively, according to the manufacturer’s instructions.

### Cell culture and treatment

A human bronchial epithelial cell line BEAS-2B (American Type Culture Collection, Manassas, VA, USA) was cultured in bronchial epithelial growth medium (BEGM) BulletKit (Lonza, Anaheim, CA, USA) at 37 °C in a 5% CO_2_ atmosphere.

To assess whether PX can cause cytotoxicity in BEAS-2B cells, BEAS-2B cells were treated with different concentrations of PX (10, 20, 40, and 80 μg/ml) for 24 h, and then cell viability and cell death were tested by MTT assay and FDA staining kit.

To investigate whether PX can inhibit LPS-induced ferroptosis and inflammation via Keap1-Nrf2/HO-1 pathway, BEAS-2B cells were co-treated with different concentrations of PX (10, 20, and 40 μg/ml), and the HO-1 inhibitor tin protoporphyrin (SnPP, 1 mM; Porphyrin Products, Logan, Ut), or Nrf2 inhibitor ML385 (5 μM; MedChem Express, New Jersey, USA) in the presence of LPS (10 μg/ml) for 24 h. Then cells and culture supernatants were collected used for ferroptosis and inflammation detection.

To investigate the relationship between ferroptosis and inflammation, the cells were treated with ferroptotic inhibitor Fer-1 (0.1 μM; Sigma-Aldrich) or deferoxamine (DFO, 10 μM; Sigma-Aldrich), or ferroptotic inducer Fe (5 M) or RAS selective lethal 3 (RSL3, 0.2 μM; Selleck Chemicals, Houston, TX, USA) in the presence of LPS (10 μg/ml) for 24 h. Then cells and culture supernatants were harvested and used for ferroptosis and inflammation detection.

### Cell viability determination

MTT assay was performed to detect cell viability. Briefly, cells were cultured under the indicated conditions. At the indicated time, 20 μl of the MTT solution (5 mg/ml) was added to each well for a 4 h co-incubation at 37 °C. The supernatant was removed, and DMSO (150 µl/well) was added to dissolve the insoluble formazan products. Absorbance at wavelength 570 nm was detected with a microplate reader (Molecular Devices, Sunnyvale, CA, USA).

### Cell death determination

Cell death was assessed by FDA staining kit (AAT Bioquest, Sunnyvale, CA, USA) in accordance with the manufacturer’s instructions.

### RT-PCR

According to our previous report [25], total RNA was extracted from lung tissues and cultured cells by using Trizol reagent (Invitrogen, Carlsbad, CA, USA) according to the instructions of the manufacturer. The cDNA was synthesized by using Reverse Transcription Kit (Takara, Dalian, China), and then PCR was performed on SmartCycler^®^ II System (Cepheid Inc., Sunnyvale, CA, USA). GAPDH was used as a reference. The relative quantification of the transcripts was calculated by using the 2^−ΔΔCt^ method.

### Western blot assay

Western blot assay was conducted as previously described [26]. The primary antibodies against GPX4 (Abcam, Cambridge, MA, USA), Keap1 (Abcam), Nrf2 (Abcam), HO-1 (Abcam) and GAPDH (Sigma-Aldrich), and horseradish peroxidase-conjugated secondary antibody (Abcam) were employed in this assay.

### Statistical analysis

All statistical analyses were conducted on GraphPad Prism 8. The data were presented as the mean ± standard deviation (SD) taken from at least three independent experiments. The comparisons between groups were performed using Student’s *t* test or ANOVA, and *P *<0.05 was regarded as statistically significant.

## Results

### Successful establishment of LPS-induced ALI in vivo

**Fig. 1:**
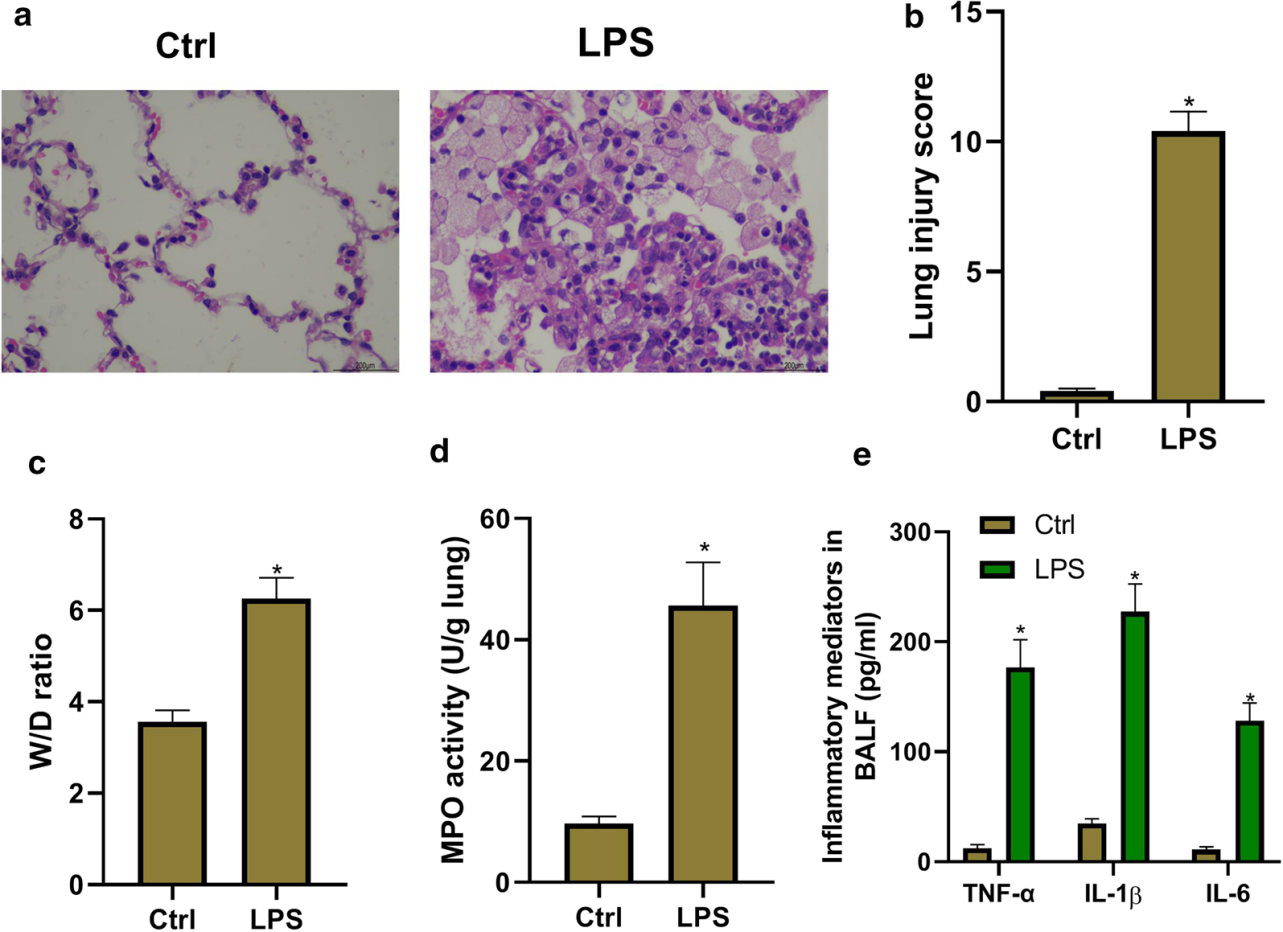
Successful establishment of LPS-induced acute lung injury in vivo. The mice are anesthetized with 30 mg/kg of pentobarbital sodium, and then LPS (10 µg/mouse) or stroke-physiological saline solution (SPSS) is injected into the trachea. Then bronchoalveolar lavage fluid (BALF) and lung tissue are collected used as the following detections. **a** The lung histological changes are assessed by haematoxylin and eosin (H&E) staining. **b** The lung injury scores are assessed by a blinded pathologist. **c** The lung Wet/Dry (W/D) weight ratio. **d** Myeloperoxidase (MPO) activity in lung tissues detected by a MPO Detection Kit. **e** Pro-inflammatory cytokines TNF-α, IL-1β, and IL-6 level in BALF determined by ELISA kits. *P < 0.05 vs. ctrl group

LPS intratracheal injection was used to establish murine ALI model. To confirm that ALI model was successfully established, pathological changes of the lung were assessed via H&E staining and lung injury score. As shown in Fig. 1a, LPS treatment induced significantly pathological changes in the lung tissues, including destroyed pulmonary architecture, thickened alveolar septa, notable inflammatory cell infiltration, alveolar hemorrhage, and interstitial and intra-alveolar edema. The lung injury scores were assessed by a blinded pathologist. Obviously, LPS group had severe lung injury (Fig. 1b). Non-cardiogenic pulmonary edema was another critical feature of ALI. Pulmonary edema was assessed through lung W/D ratio, and total protein concentrations in BALF. As shown in Fig. 1c and Table 1, LPS group had the significantly higher W/D ratio and total protein concentration in BALF than the control group. MPO activity was used to assess the activation and accumulation of neutrophils in the lung tissues. The results showed that LPS significantly increased MPO activity (Fig. 1d). Expectedly, there were more total cells in LPS group, and the proportion of neutrophils increased compared with the control group (Table 1). In addition, pro-inflammatory cytokines TNF-α, IL-1β, and IL-6 level in BALF were determined. Exposure to LPS caused a significant increase in TNF-α, IL-1β, and IL-6 level in BALF (Fig. 1e). Taken together, ALI model is successfully established by LPS intratracheal injection.

**Table 1:** The total protein, total cells and neutrophil percentage in BALF in different groups

Group	Total protein (mg/ml)	Total cell number (10^5^)	Neutrophil percentage (%)
Control	0.2 ± 0.02	0.85 ± 0.12	4.32 ± 0.55
LPS	0.95 ± 0.11^*^	4.58 ± 0.45^*^	46.8 ± 5.21^*^
PX+LPS	0.45 ± 0.05^#^	2.51 ± 0.23^#^	17.23 ± 1.82^#^
Fe+LPS	1.32 ± 0.22^#^	5.94 ± 0.50^#^	65.78 ± 6.53^#^
PX+Fe+LPS	0.51 ± 0.1^&^	3.25 ± 0.42^&^	25.64 ± 2.98^&^

The data are expressed as the mean ± SEM

BALF: bronchoalveolar lavage fluid; PX: panaxydol

Compare with control group,^*^P < 0.05; compare with LPS group, ^*#*^*P *< 0.05; and compare with Fe+LPS group, ^*&*^*P* < 0.05

### Ferroptosis is increased in LPS-induced ALI

**Fig. 2:**
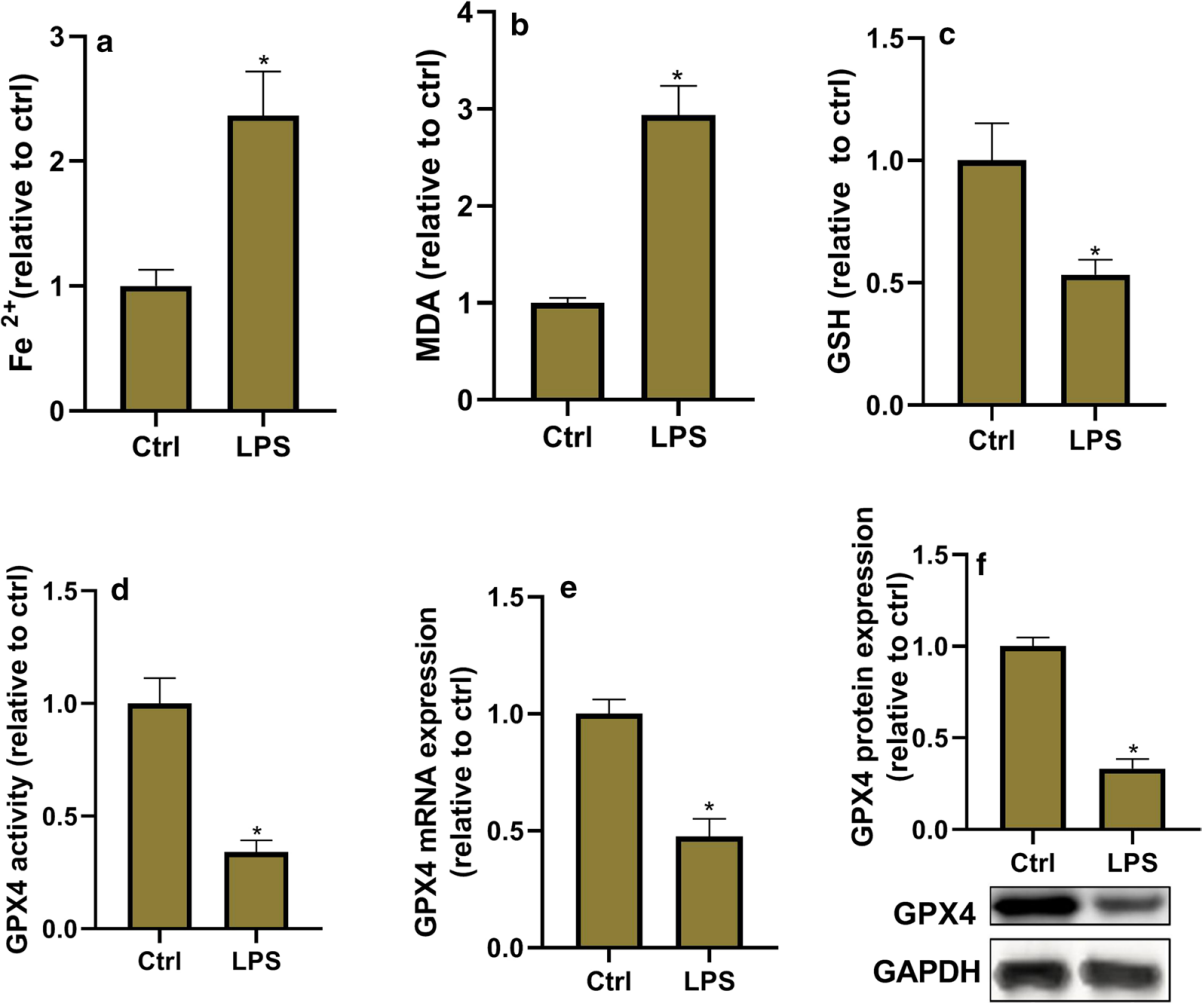
Ferroptosis is increased in LPS-induced acute lung injury. The ferroptosis level is evaluated in lung tissues via detection of the biomarkers of ferroptosis. **a** Fe^2+^ level determined using Iron Assay Kit. **b** Malondialdehyde (MDA) level detected via the MDA Assay Kit. **c** Glutathione (GSH) level detected by GSH assay kit. **d** Glutathione Peroxidase 4 (GPX4) activity measured by GPX4 ELISA kit. **e** GPX4 mRNA expression determined by RT-PCR. **f** GPX4 protein expression detected by western blot assay. *P < 0.05 vs. ctrl group

The ferroptosis level was evaluated in lung tissues via detection of ferroptosis biomarkers Fe^2+^, MDA and GSH level, and GPX4 activity, mRNA and protein expression. Ferroptosis is believed to be caused by iron accumulation and lipid peroxidation. MDA is a final product of lipid peroxidation. GPX4, a phospholipid hydroperoxidase, possesses the unique capability to suppress lipid peroxidation. GSH is an antioxidant compound, and its depletion directly activates lipoxygenases and inhibits GPX4 activity to induce lipid peroxidation. As shown in Fig. 2, MDA and Fe^2+^ levels significantly increased, and GSH level, and GPX4 activity, mRNA and protein expression significantly decreased in LPS group compared with the control group. These data suggested that LPS treatment promotes ferroptosis in lung tissues.

### PX inhibits LPS-induced ALI and ferroptosis in vivo

**Fig. 3:**
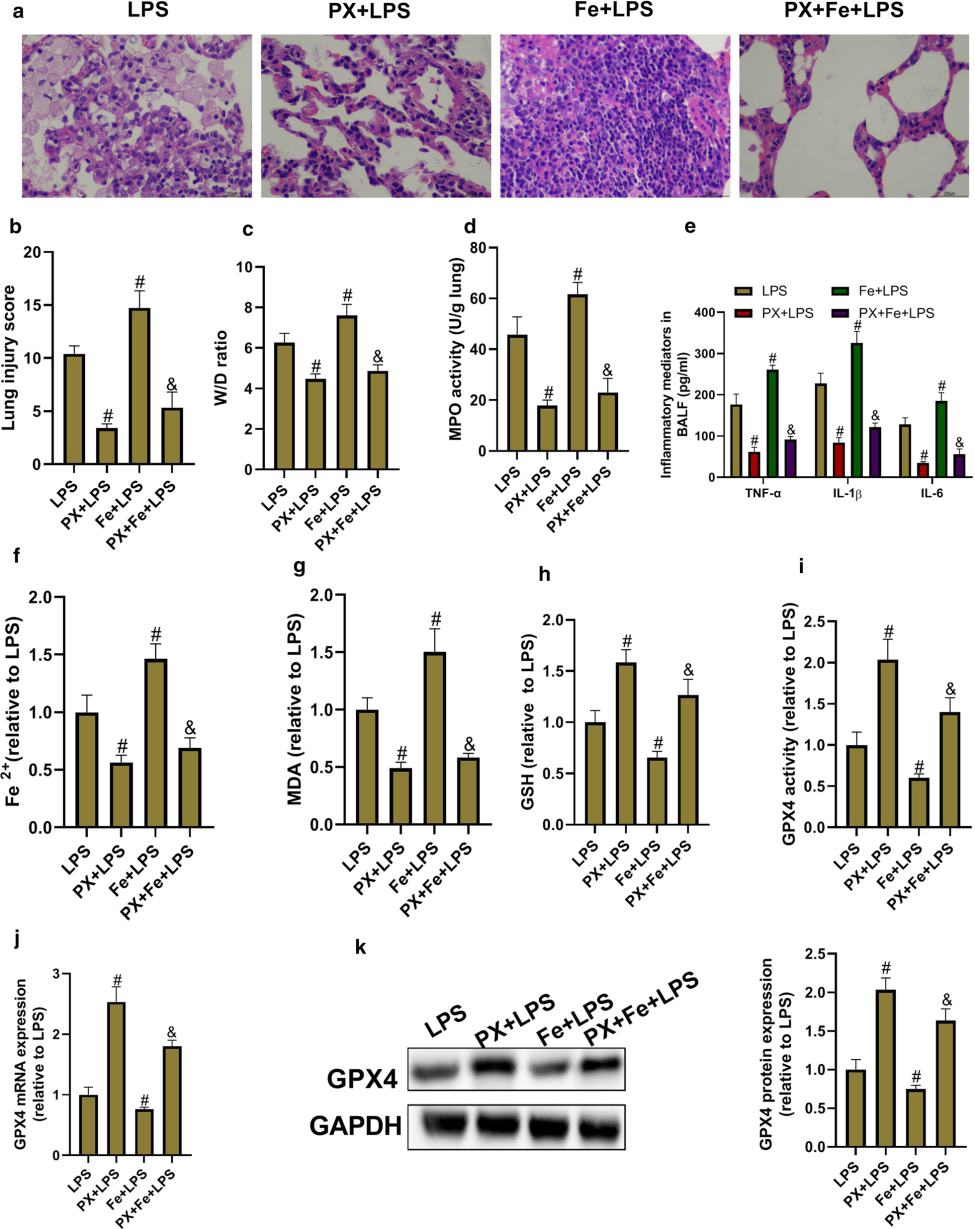
Panaxydol (PX) inhibits LPS-induced ALI and ferroptosis in vivo. Fe-citrate (III) (Fe) is dissolved in stroke-physiological saline solution (SPSS). PX is dissolved in dimethyl sulfoxide (DMSO), and further diluted in SPSS. Intravenous injection of Fe or/and intraperitoneal injection of PX are performed from day 0 to day 2. At 1 h after the final Fe and PX treatment, the mice are anesthetized with 30 mg/kg of pentobarbital sodium, and then LPS (10 µg/mouse) or SPSS is injected into the trachea. Then BALF and lung tissue are collected used as the following detections. **a** The lung histological changes are assessed by H&E staining. **b** The lung injury scores. **c** The lung W/D weight ratio. **d** MPO activity in lung tissues. **e** TNF-α, IL-1β, and IL-6 level in BALF. **f** Fe^2+^ level. **g** MDA level. **h** GSH level. **i** GPX4 activity. **j** GPX4 mRNA expression. **k** GPX4 protein expression. ^*#*^*P *< 0.05 vs. LPS group, and ^*&*^*P *< 0.05 vs. Fe+LPS group

The therapeutic action of PX against LPS-induced ALI and ferroptosis was evaluated in vivo. As shown in Fig. 3a and b, PX treatment significantly ameliorated LPS-induced pathological changes in the lung tissues; the addition of Fe aggravated LPS-induced pathological changes; and co-treatment with PX and Fe obviously relieved the effects induced by Fe. Lung injury scores were assessed. Not surprisingly, Fe+LPS group had the severest lung injury, followed by LPS group, PX+Fe+LPS group, and PX+LPS group. In addition, pulmonary edema (Fig. 3c, Table 1), inflammation (Fig. 3d and e, Table 1) and ferroptosis (Fig. 3f–k) were also assessed in all groups. Fe+LPS group had the severest pulmonary edema, inflammation, and ferroptosis, followed by LPS group, PX+Fe+LPS group, and PX+LPS group. These data indicated that PX inhibits LPS-induced ALI and ferroptosis. Additionally, in view that Fe, a promoter of ferroptosis, significantly aggravated LPS-induced ALI, and PX treatment significantly relieved the effects induced by Fe, it tempted us to speculate that PX ameliorates LPS-induced ALI via inhibition of ferroptosis.

### PX inhibits LPS-induced ferroptosis and inflammation in vitro

**Fig. 4:**
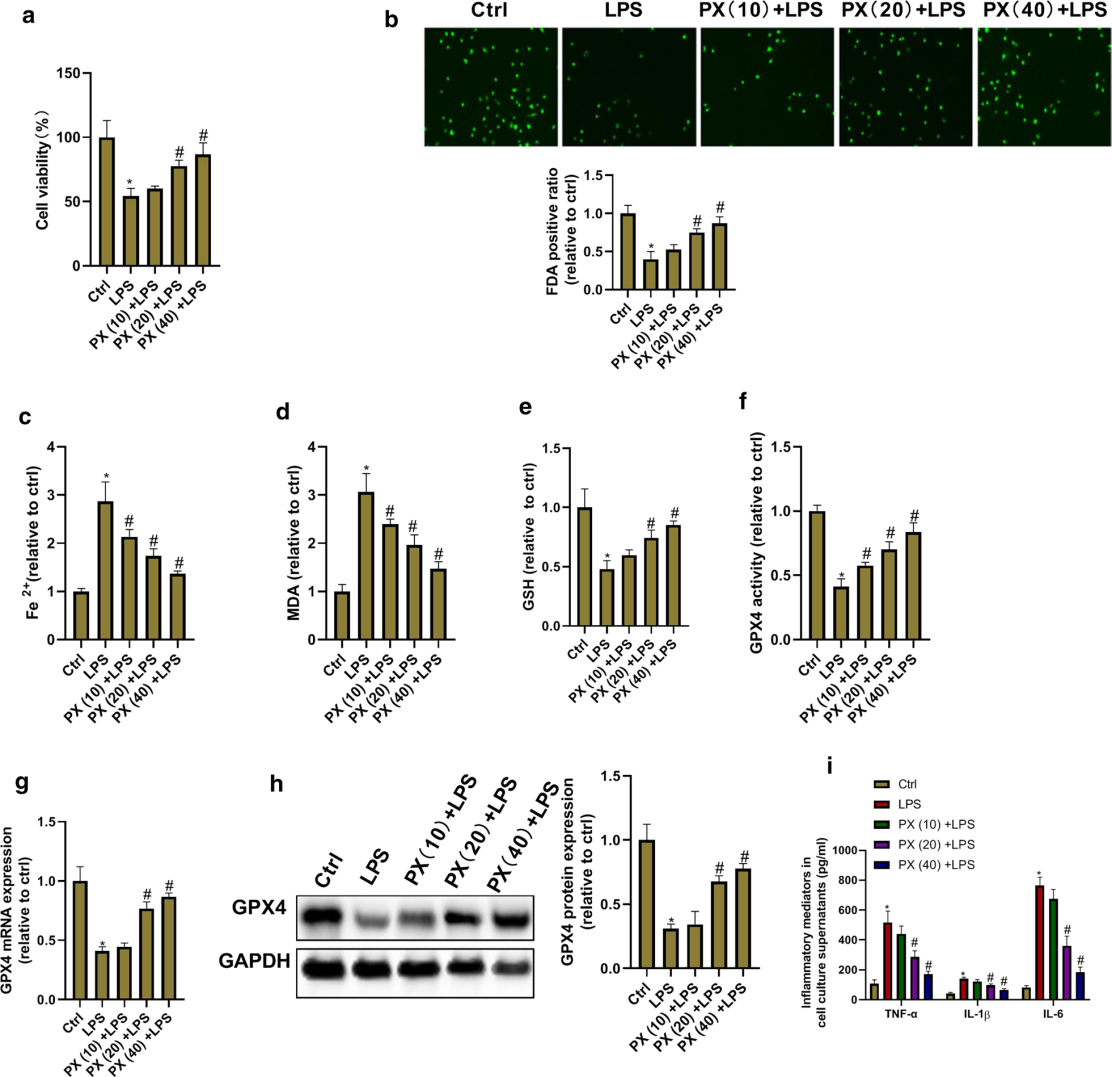
PX inhibits LPS-induced ferroptosis and inflammation in vitro. BEAS-2B cells are treated with different concentrations of PX (10, 20, and 40 μg/ml) in the presence of LPS (10 μg/ml) for 24 h. Then cells and culture supernatants are collected used for ferroptosis and inflammation detection. **a** Cell viability determined by MTT assay. **b** Cell death detected by FDA staining kit. **c** Fe^2+^ level determined using Iron Assay Kit. **d** MDA level detected via the MDA Assay Kit. **e** GSH level detected by GSH assay kit. **f** GPX4 activity measured by GPX4 ELISA kit. **g** GPX4 mRNA expression detected by RT-PCR. **h** GPX4 protein expression detected by western blot assay. **i** TNF-α, IL-1β, and IL-6 level in cell culture supernatants determined via ELISA Kits. ^*^P <0.05 vs. ctrl group, and ^*#*^*P *<0.05 vs. LPS group

We next sought to explore the function of PX in LPS-induced injury of bronchial epithelial cells (BEAS-2B cells). We first investigated whether PX can cause cytotoxicity in BEAS-2B cells under normal culture conditions. As shown in Additional file 1: Fig. S1, PX did not cause cytotoxicity in BEAS-2B cells at concentrations up to 40 μg/ml. But at high concentration of 80 μg/ml, PX not only decreased the cell viability, but also caused the cell death (green fluorescence representing living cells). Thus, we chose PX concentration of 10, 20 and 40 μg/ml to investigate whether PX mediates LPS-induced ferroptosis and inflammation in vitro. As shown in Fig. 4a–h, LPS induced ferroptotic phenotypes including decreased cell viability, increased cell death, Fe^2+^ accumulation, excessive lipid peroxide MDA production, and ROS scavenging enzyme GSH and GPX4 deletion. There existed a dose-dependent inhibition of PX on LPS-induced ferroptotic phenotypes. In addition, PX effectively inhibited LPS-induced TNF-α, IL-1β, and IL-6 production in a concentration-dependent way. These data suggested that PX inhibits LPS-induced ferroptosis and inflammation in vitro.

### Ferroptosis mediates inflammation in LPS-treated BEAS-2B cells

**Fig. 5:**
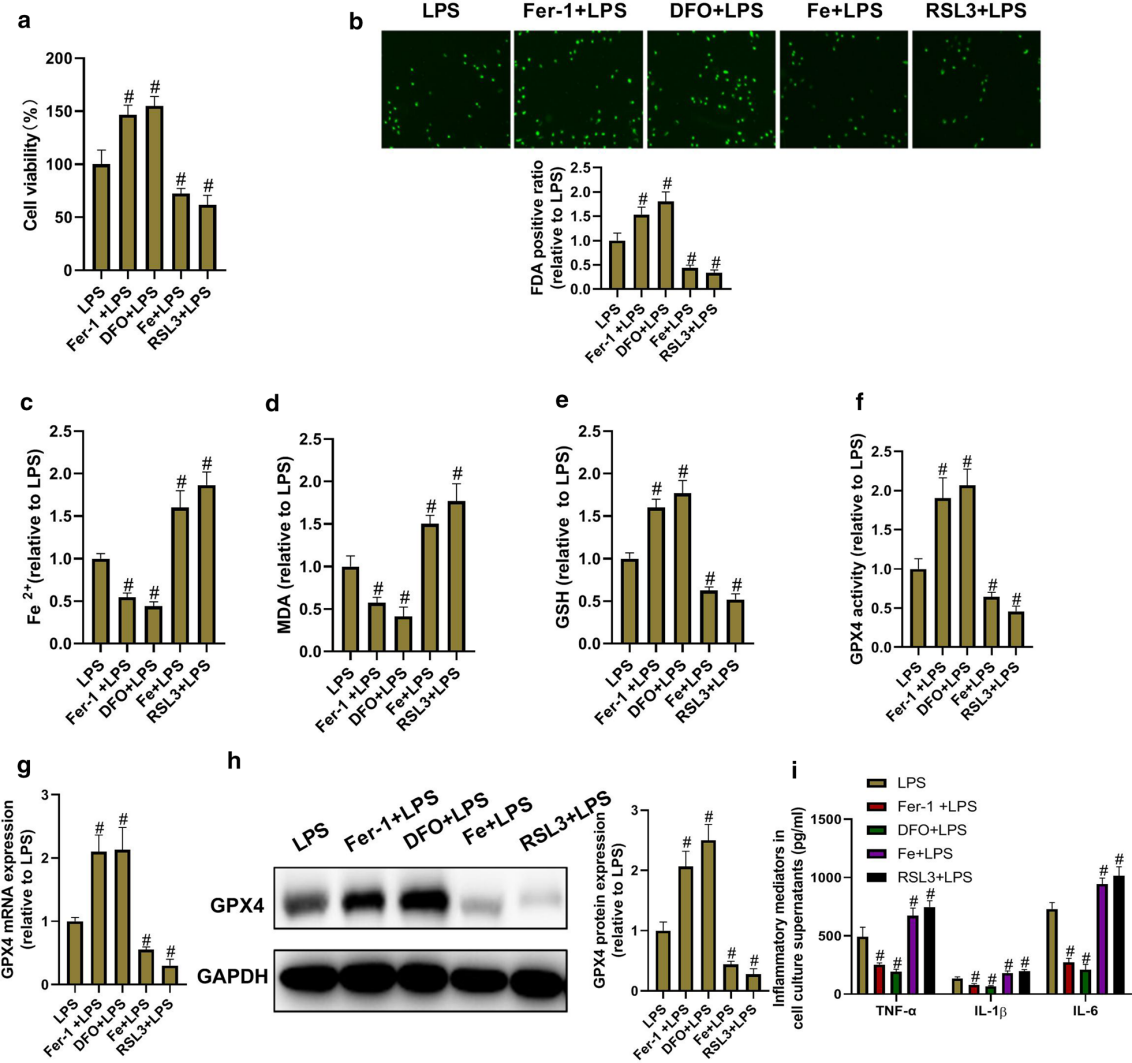
Ferroptosis mediates inflammation in LPS-treated BEAS-2B cells. The cells are treated with ferroptotic inhibitor Fer-1 (0.1 μM) or DFO (10 μM), or ferroptotic inducer Fe (5 M) or RSL3 (0.2 μM) in the presence of LPS for 24 h. Then ferroptosis and inflammation are assessed. **a** Cell viability determined by MTT assay. **b** Cell death detected by FDA staining kit. **c** Fe^2+^ level determined using Iron Assay Kit. **d** MDA level detected via the MDA Assay Kit. **e** GSH level detected by GSH assay kit. **f** GPX4 activity measured by GPX4 ELISA kit. **g** GPX4 mRNA expression decided by RT-PCR. **h** GPX4 protein expression detected by western blot assay. **i** TNF-α, IL-1β, and IL-6 level in cell culture supernatants determined via ELISA Kits. ^*#*^*P *<0.05 vs. LPS group

To investigate whether PX ameliorates LPS-induced inflammation in vitro and in vivo via inhibition of ferroptosis, the relationship between ferroptosis and inflammation was investigated. The cells were treated with ferroptotic inhibitor Fer-1 or DFO, or ferroptotic inducer Fe or RSL3 in the presence of LPS for 24 h. Then ferroptosis and inflammation were assessed. As shown in Fig. 5, Fer-1 and DFO significantly inhibited LPS-induced ferroptosis and inflammation, and Fe and RSL3 significantly aggravated LPS-induced ferroptosis and inflammation. These data suggested that ferroptosis mediates inflammation in LPS-treated BEAS-2B cells, and PX might ameliorate LPS-induced inflammation via inhibition of ferroptosis.

### PX upregulates Keap1-Nrf2/HO-1 pathway

**Fig. 6:**
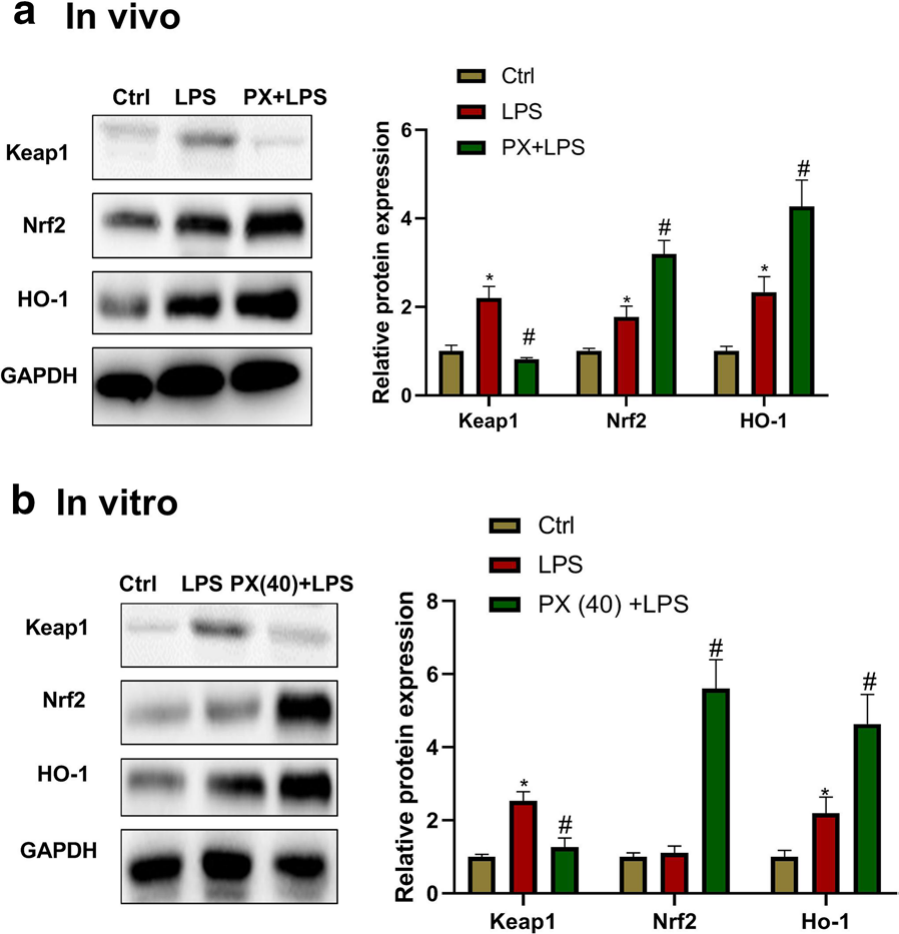
PX upregulates Keap1-Nrf2/HO-1 pathway. Keap1, Nrf2 and HO-1 protein expression in lung tissues (**a**) and cultured cells (**b**) detected by western blot assay. ^*^P <0.05 vs. ctrl group, and ^*#*^*P *< 0.05 vs. LPS group

To investigate whether PX modulates Keap1-Nrf2/HO-1 signaling in LPS-induced ALI in vivo and in vitro, the protein expression of Keap1, Nrf2, and HO-1 was detected. Keap1 is the inhibitor of Nrf2, and HO-1 is the downstream gene of Nrf2. As shown in Fig. 6a and b, compared with the control group, LPS treatment increased Keap1, Nrf2, and HO-1 protein in vivo and in vitro, though no significant difference in Nrf2 expression in vitro. PX treatment significantly decreased Keap1 expression, and increased Nrf2 and HO-1 expression in comparison with LPS group, which suggested that PX upregulates Keap1-Nrf2/HO-1 pathway.

### PX functions via Keap1-Nrf2/HO-1 pathway

**Fig. 7:**
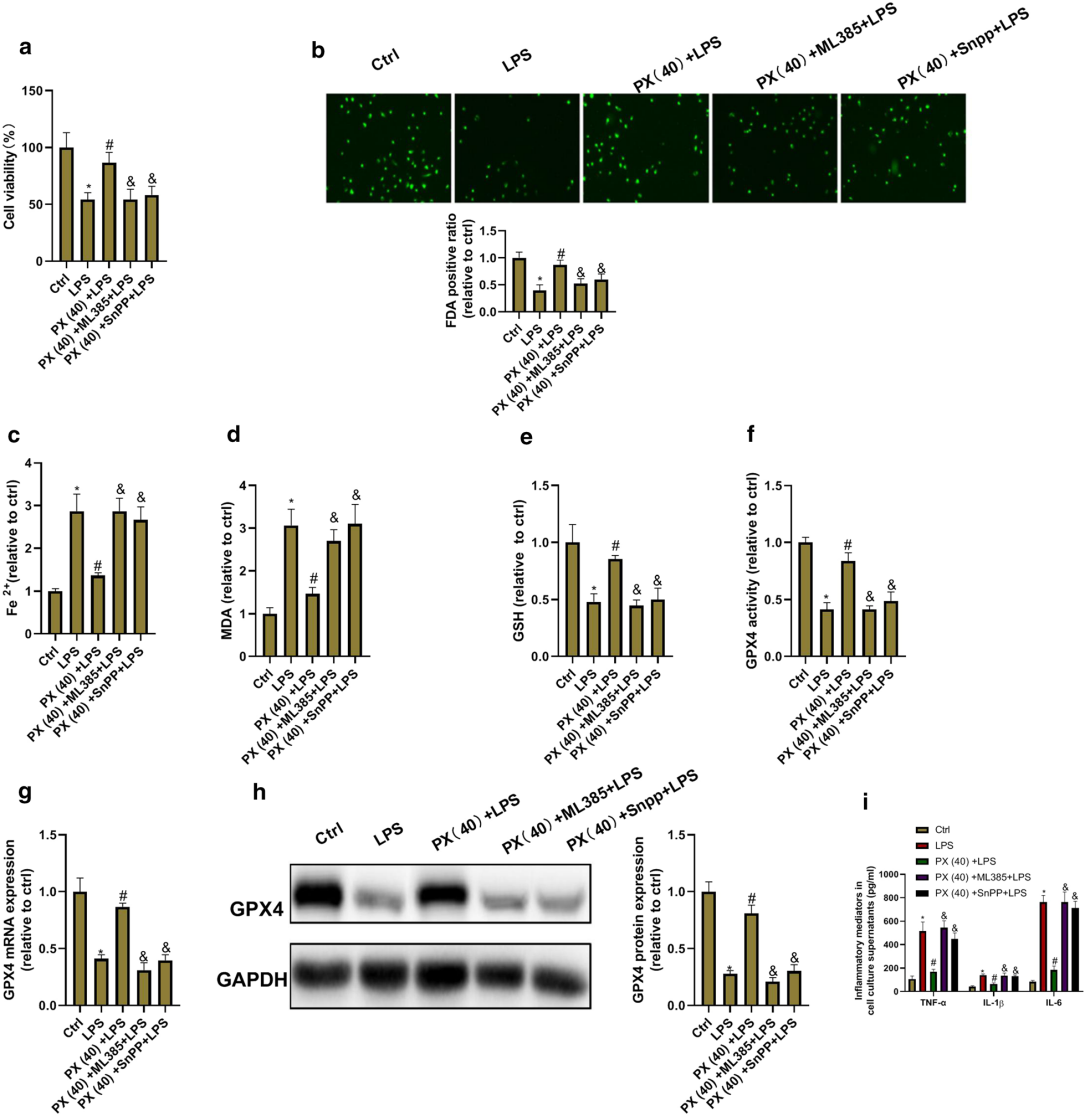
PX inhibits ferroptosis via Keap1-Nrf2/HO-1 pathway. BEAS-2B cells are co-treated with 40 μg/ml of PX and the HO-1 inhibitor SnPP or Nrf2 inhibitor ML385 in the presence of LPS for 24 h. Then cell ferroptosis and inflammation are detected. **a** Cell viability determined by MTT assay. **b** Cell death detected by FDA staining kit. **c** Fe^2+^ level determined using Iron Assay Kit. **d** MDA level detected via the MDA Assay Kit. **e** GSH level detected by GSH assay kit. **f** GPX4 activity measured by GPX4 ELISA kit. **g** GPX4 mRNA expression determined by RT-PCR. **h** GPX4 protein expression detected by western blot assay. **i** TNF-α, IL-1β, and IL-6 level in cell culture supernatants determined via ELISA Kits. ^*^P < 0.05 vs. ctrl group, ^*#*^*P *< 0.05 vs. LPS group, and ^*&*^*P *< 0.05 vs. PX(40) + LPS group

We further investigated whether PX functions via Keap1-Nrf2/HO-1 signaling. BEAS-2B cells were co-treated with 40 μg/ml of PX and the HO-1 inhibitor SnPP or Nrf-2 inhibitor ML385 in the presence of LPS for 24 h. Then cell ferroptosis and inflammation were detected. As shown in Fig. 7, SnPP and ML385 significantly abolished the anti-ferroptotic and anti-inflammatory functions of PX in LPS-treated cells. These data indicated that PX functions via Keap1-Nrf2/HO-1 pathway.

## Discussion

ALI/ARDS causes severe lung diseases or the pulmonary presentation of multiple organ dysfunction syndromes (MODS), and induces uncontrolled and self-amplified pulmonary inflammation [27–29]. Inflammation plays a pivotal role in ALI/ARDS, and could directly or indirectly result in the injuries of alveolar epithelium and microvascular endothelium in lung, which are the primary source of pathogenesis of ALI/ARDS [28, 30]. This disease has been a heavy burden for the public due to its high morbidity and mortality in critically ill patients [2]. Although great progress has been made in understanding the pathogenesis of the disease, the treatments available in clinical practice are still limited, mainly supportive treatment, such as nutritional support, mechanical ventilation, etiological treatment and comprehensive treatment to maintain fluid, electrolyte, acid and alkali balance. Therefore, it is of great value and significance to find a new drug or treatment for ALI/ARDS.

In recent years, many bioactive ingredients extracted from herbs have been reported to effectively ameliorate ALI/ARDS via different mechanisms. For example, glycyrrhizic acid inhibits LPS-induced inflammation in ALI by regulating the PI3K/AKT/mTOR pathway related autophagy [31]; absinthin attenuates LPS-induced ALI through MIP-1α-mediated inflammatory cell infiltration [32]; picrasma quassiodes exerts anti-inflammatory effects against LPS-induced ALI via the modulation of iNOS, HO-1, NF-κB and MAPK signaling [33]; emodin potently inhibits LPS-induced pulmonary inflammation and pulmonary edema via regulation of NF-κB pathway [34]; and magnoflorine protects against the production of inflammatory factors in LPS-induced ALI at least partially by inhibiting TLR4-mediated NF-κB and MAPK signaling pathways [35].

PX, isolated from the roots of Panax ginseng, has been confirmed to have great values in various human diseases, such as cancer, renal injury, and neurodegenerative disease [21–24]. However, whether PX has therapeutic potential for ALI is unclear. In this study, we explored the function of PX in LPS-induced ALI. We found that administration of PX effectively inhibited LPS-induced pulmonary pathological changes, pulmonary inflammation and edema in vivo. In addition, we also detected the effects of PX on ferroptosis. Ferroptosis occurred in LPS-induced ALI, consistent with Liu’s report [17], and PX significantly decreased LPS-induced ferroptosis. In vitro studies, PX had also been proved to have anti-inflammatory and anti-ferroptotic functions. Many studies have documented that ferroptosis contributes to the progression of ALI [14–17]. Ferroptosis is categorized as regulated necrosis, which is more immunogenic than apoptosis. So far, increasing body of evidence points that ferroptosis plays an important role in inflammation, and some ferroptosis inhibitors have been shown to play an anti-inflammatory role in certain diseases. For example, Tsurusaki et al. [36] reported that hepatic ferroptosis plays a significant role as the trigger for initiating inflammation in steatohepatitis; Zhou et al. [11] found that intestinal SIRT1 deficiency alleviates inflammation in the ethanol-induced mouse model of hepatitis by mitigating ferroptosis; and Prakash et al. [37] reported that ferroptosis mediates inflammation in lung I/R sterile injury in mice. We also investigated the relationship between ferroptosis and inflammation in LPS-induced ALI model in vitro. The results showed that ferroptosis mediated inflammation in LPS-treated BEAS-2B cells, and PX might ameliorate LPS-induced inflammation via inhibiting ferroptosis.

We further investigated the underlying mechanism by which PX functions in LPS-induced ALI. A recent study documented [24] that PX could suppress aristolochic acid-induced renal failure by suppressing oxidative stress through the activation of Keap1-Nrf2/HO-1 signaling pathway.

Keap1-Nrf2/HO-1 signaling is regarded as one of the most pivotal endogenous antioxidative stress pathway, which is the significant target for inflammation-related disorders [38, 39]. Under unstressed conditions, Nrf2 is negatively regulated by Keap1 via interaction with Nrf2 and promoting its degradation, and remains at low cellular concentrations. Once the cells are damaged, Nrf2 will overcome this suppression, translocates into the nucleus, and subsequently activates various genes, including HO-1. To date, compelling evidence indicates that HO-1 is a critical protein of ferroptosis occurrence, and many drugs inhibit ferroptosis via activation of Nrf2/HO-1 signaling. For example, gastrodin protects HT-22 cells from the ferroptosis induced by glutamate through Nrf2/HO-1 signaling pathway [40]; ginkgolide B exerts anti-ferroptosis effects by activation of Nrf2/HO-1 signaling pathway in high fat diet induced nonalcoholic fatty liver disease [41]; and proanthocyanidin inhibits ferroptosis and promotes functional recovery of spinal cord injury via Nrf2/HO-1 signaling [42]. In this study, the results showed that PX inhibited LPS-induced ferroptosis and inflammation via Keap1-Nrf2/HO-1 signaling.

## Conclusions

In summary, the major findings of this study can be summarized as follows: (1) PX inhibits LPS-induced inflammation and ferroptosis in vivo and in vitro; (2) Ferroptosis mediates inflammation in LPS-treated BEAS-2B cells, and PX might ameliorate LPS-induced inflammation via inhibition of ferroptosis; (3) The mechanisms of PX in regulation of ferroptosis and inflammation in BEAS-2B cells are via Keap1-Nrf2/HO-1 pathway; and (4) Selective inhibition of Keap1-Nrf2/HO-1 pathway significantly attenuates LPS-induced inflammation and ferroptosis in vitro. These findings suggest that PX is a promising novel therapeutic candidate for ALI.

## Supplementary Information

**Additional file 1.**

## Data Availability

The datasets supporting our findings are presented in the article.

## Abbreviations

ALI: Acute lung injury
ARDS: Acute respiratory distress syndrome
PX: Panaxydol
LPS: Lipopolysaccharide
ECM: Extracellular matrix
I/R: Ischemia/reperfusion
Fer-1: Ferrostatin-1
MODS: Multiple organ dysfunction syndromes
W/D: Wet/dry
H&E: Hematoxylin and eosin
MDA: Malondialdehyde
GPX4: Glutathione Peroxidase 4
GSH: Glutathione
DFO: Deferoxamine
RSL3: RAS selective lethal 3
