# Supplementary figures and images for: Panaxydol attenuates ferroptosis against LPS-induced acute lung injury in mice by Keap1-Nrf2/HO-1 pathway
Source: J Transl Med. 2021 Mar 2;19:96. doi: 10.1186/s12967-021-02745-1 (PMC7927246; doi:10.1186/s12967-021-02745-1)

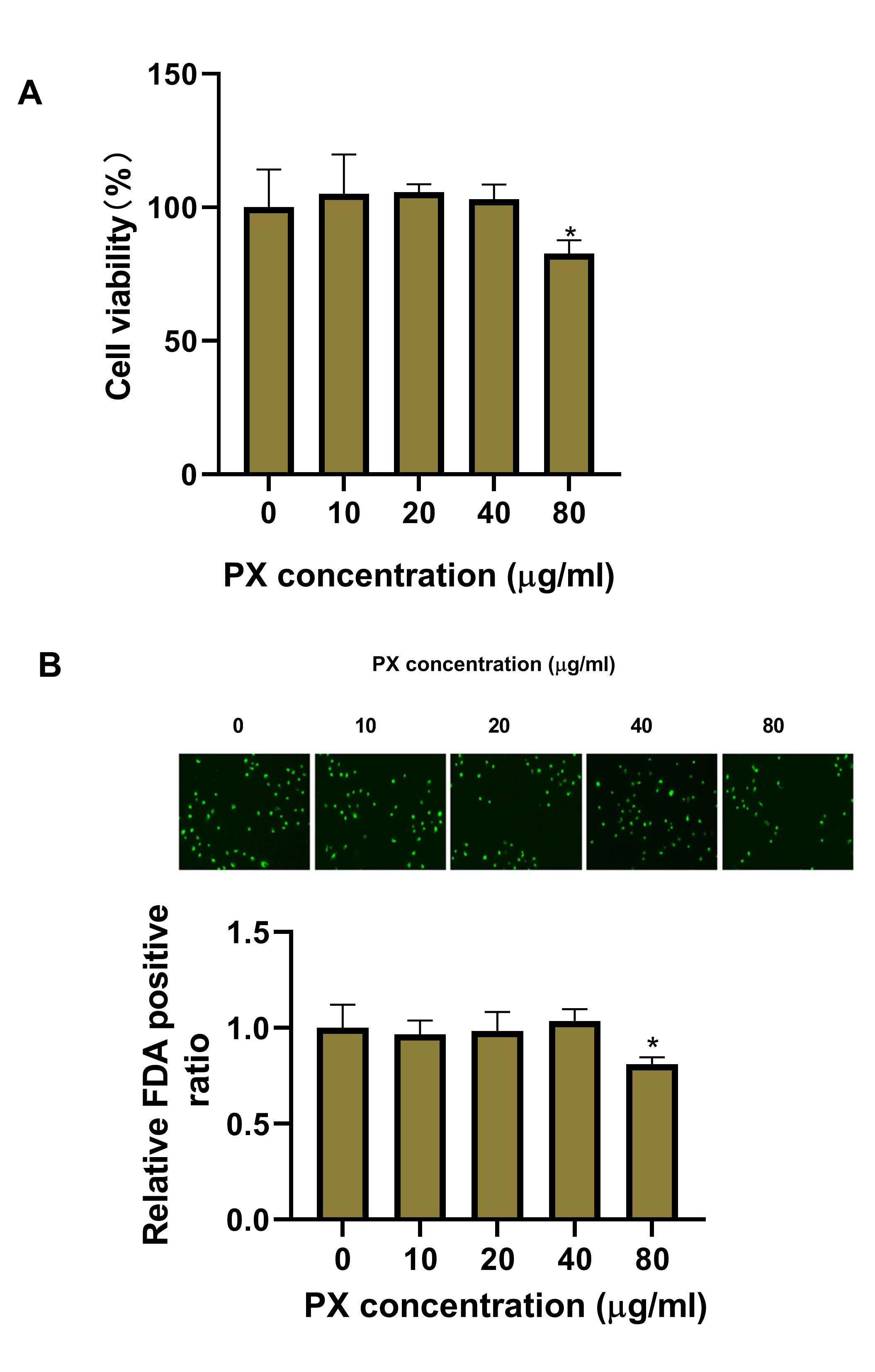

Supplement: Supplementary file 1 — Additional file 1. [file 12967_2021_2745_MOESM1_ESM.jpg]
